# Extraintestinal Manifestations in Induced Colitis: Controversial Effects of *N*-Acetylcysteine on Colon, Liver, and Kidney

**DOI:** 10.1155/2023/8811463

**Published:** 2023-08-05

**Authors:** Amylly Sanuelly da Paz Martins, Kívia Queiroz de Andrade, Orlando Roberto Pimentel de Araújo, Glenn Côsallin Melquiades da Conceição, Amanda da Silva Gomes, Marília Oliveira Fonseca Goulart, Fabiana Andréa Moura

**Affiliations:** ^1^Doctoral Program of the Northeast Biotechnology Network, Federal University of Alagoas, Maceió 57072-970, Alagoas, Brazil; ^2^College of Nutrition, Federal University of Alagoas, Maceió 57072-970, Alagoas, Brazil; ^3^Institute of Chemistry and Biotechnology, Federal University of Alagoas, Maceió 57072-970, Alagoas, Brazil; ^4^Institute of Biological and Health Sciences, Federal University of Alagoas, Maceió 57072-970, Alagoas, Brazil; ^5^College of Medicine, Federal University of Alagoas, Maceió 57072-970, Alagoas, Brazil

## Abstract

Ulcerative colitis (UC) is a chronic and recurrent inflammatory bowel disease (IBD) characterized by continuous inflammation in the colonic mucosa. Extraintestinal manifestations (EIM) occur due to the disruption of the intestinal barrier and increased permeability caused by redox imbalance, dysbiosis, and inflammation originating from the intestine and contribute to morbidity and mortality. The aim of this study is to investigate the effects of oral *N*-acetylcysteine (NAC) on colonic, hepatic, and renal tissues in mice with colitis induced by dextran sulfate sodium (DSS). Male Swiss mice received NAC (150 mg/kg/day) in the drinking water for 30 days before and during (DSS 5% v/v; for 7 days) colitis induction. On the 38^th^ day, colon, liver, and kidney were collected and adequately prepared for the analysis of oxidative stress (superoxide dismutase (SOD), catalase (CAT), glutathione reduced (GSH), glutathione oxidized (GSSG), malondialdehyde (MDA), and hydrogen peroxide (H_2_O_2_)) and inflammatory biomarkers (myeloperoxidase (MPO) –, tumor necrosis factor alpha – (TNF-*α*, and interleukin-10 (IL-10)). In colon, NAC protected the histological architecture. However, NAC did not level up SOD, in contrast, it increased MDA and pro-inflammatory effect (increased of TNF-*α* and decreased of IL-10). In liver, colitis caused both oxidative (MDA, SOD, and GSH) and inflammatory damage (IL-10). NAC was able only to increase GSH and GSH/GSSG ratio. Kidney was not affected by colitis; however, NAC despite increasing CAT, GSH, and GSH/GSSG ratio promoted lipid peroxidation (increased MDA) and pro-inflammatory action (decreased IL-10). Despite some beneficial antioxidant effects of NAC, the negative outcomes concerning irreversible oxidative and inflammatory damage in the colon, liver, and kidney confirm the nonsafety of the prophylactic use of this antioxidant in models of induced colitis, suggesting that additional studies are needed, and its use in humans not yet recommended for the therapeutic routine of this disease.

## 1. Introduction

Ulcerative colitis (UC) is a chronic and recurrent inflammatory bowel disease (IBD) characterized by continuous inflammation in the colonic mucosa that leads to the appearance of typical clinical manifestations: bloody diarrhea and abdominal pain [1]. The incidence and prevalence of UC have increased in recent years, especially in developed regions such as the United States, Canada, and Europe, with a notable expansion in developing countries [2].

The etiology of UC is not fully understood but is known to be multifactorial, involving complex interactions between genetic, environmental, and immunological factors, beyond redox imbalance. Under physiological conditions, the human body has a potent antioxidant defense system that comprises enzymes such as superoxide dismutase (SOD), catalase (CAT), and glutathione peroxidase (GPx), and low-molecular-weight compounds, such as glutathione (GSH) and other thiols, ascorbic acid, and *α*-tocopherol [3]. However, in UC, excessive production of reactive oxygen and nitrogen species (RONS), associated with a decrease in total antioxidant capacity, occurs, leading to a higher secretion of pro-inflammatory cytokines. Tumor necrosis factor alpha (TNF-*α*), interleukin (IL)-6, and IL-1*β* levels are enhanced, while decreases in the levels of anti-inflammatory cytokines, such as IL-10, had contributed to the progression and/or maintenance of oxidative damage to the colonic mucosa [4, 5].

The occurrence of extraintestinal manifestations (EIM) is reported in ∼30%–50% of IBD patients, and although their etiologies are not yet completely clarified, redox imbalance, dysbiosis, and inflammation originating from the intestine are involved in their appearance and contribute to morbidity and mortality in these patients [6]. Intestinal barrier dysfunction and its increased permeability allow luminal antigens to diffuse via the bloodstream and cause inflammatory responses in other organs [7]. Population-based studies have reported that the emergence of EIM is directly associated with the extension of colonic injury [8]. The most common EIM are hepatobiliary, osteoarticular, dermatological, and ophthalmological; however, although less frequent, the involvement of other organs, such as the brain, heart, and kidney, has been described [7].

Despite the high prevalence and impact on patients with IBD, EIM are still poorly studied both in the fundamental areas of research and in clinical trials. The absence of answers about the etiology of EIM, as well as the impact of conventional and unconventional treatments of IBD on these manifestations, brings some knowledge' gaps concerning the full effectiveness of these therapies. In this context, in 2019, Hedin et al. [9], sought to expand the knowledge brought by the first European Evidence-based Consensus on EIM in IBD, published in 2016. The authors, at the end of several topics, brought relevant questions that need still to be answered. The scientific literature needs to strive to understand them, aiming to adequately treat these patients [9].

Current IBD medical therapy includes the use of aminosalicylates, corticosteroids, immunosuppressants, and biological therapy. However, due to their limited effectiveness and adverse effects, several alternative treatments are being investigated, such as the administration of *N*-acetylcysteine (NAC) [11].

NAC is a reducing agent and a cysteine-producing prodrug that functions as a GSH precursor [12]. In addition, NAC is an important scavenger of RONS and plays a role as a metal chelator and anti-inflammatory agent via suppression of nuclear factor-activated B-cell kappa (NF-*κ*B) with consequent reduction of TNF-*α*, IL-6, and IL-1*β* and inhibition of chemotaxis in neutrophils and macrophages [13]. Due to its *N*-acetyl portion, NAC is more resistant to oxidation in a physiological environment and has lower toxicity, because when orally ingested, it is slowly absorbed, leading to a bioavailability of 9% and 4% for total and reduced NAC, respectively [14, 15]. Additionally, NAC has already been tested in patients with UC, in a pilot, placebo-controlled study [16] and, more recently [17], in a randomized, double-blind controlled clinical trial. In both cases, NAC was administered in combination with mesalamine.

Despite having already been tested in humans, the effect of NAC on organs and tissues other than the colon in IBD is a gap in the scientific community. In this context, this paper proposes to discuss two questions launched by the authors: (1) could further animal models with intestinal inflammation and EIM (including sites other than joints) be developed? (2) can animal models be used to elucidate the temporal relationship between intestinal disease and development of EIM? Additionally in this study, we investigate the potential role of NAC in a dextran sulfate sodium (DSS)-induced mice model of IBD and its effects on colon, liver, and kidney.

## 2. Materials and Methods

### 2.1. Chemicals

DSS (MW 36,000–40,000 Da) was purchased from MP Biomedicals® (Santa Ana, CA, USA). The SOD Assay Kit was from Sigma–Aldrich® (St. Louis, USA). Cytokine kits were obtained from PeproTech® (PeproTech Brasil FUNPEC, Ribeirão Preto, SP, BR), protease inhibitor cocktail tablets were obtained from Roche® (Germany), and radioimmunoprecipitation assay (RIPA) buffer was obtained from Cell Signaling® (Danvers, MA, USA). All other chemicals and enzymes were purchased from Sigma–Aldrich® (St. Louis, MO, USA).

### 2.2. Equipment

The equipment used included a bio-freezer VIP Series (Sanyo), a ultaviolet/visible spectrophotometer (Thermo Scientific), a magnetic stirrer AP 55 (Phoenix), an Olympus BX51 (Nikon) optical microscope connected to a DP70 digital camera system (Olympus, Tokyo, Japan), and a high-performance liquid chromatograph (HPLC) coupled to a ultaviolet detector (Shimadzu®, serial no. L201550).

### 2.3. Animals

The experimental protocol was approved by the Institutional Animal Ethics Committee/Federal University of Alagoas (Universidade Federal de Alagoas; IAEC/UFAL; 45/2016) and was developed according to the Guide for the Care and Use of Laboratory Animals. Eighteen male Swiss mice, 8 weeks old (average body mass: 31 g), from the Central Animal Farm of the UFAL (BIOCEN/UFAL) were used and kept in polyethylene cages (six animals per cage) at the Sectorial Bioterium of the Faculty of Nutrition at UFAL, under adequate temperature (24 ± 1°C), humidity (50% ± 10%), and light (12/12 hr light/dark cycle) conditions. Commercial feed (Rhoster®—SP, Brazil) and water were provided *ad libitum*. Initially, all animals underwent an acclimatization period for 1 week, where food and water intake were evaluated, considering the average of each group. Thus, the administered dose of NAC was based on the group's average water intake, which was changed daily.

### 2.4. Experimental Design and Colitis Induction

**Figure 1:**
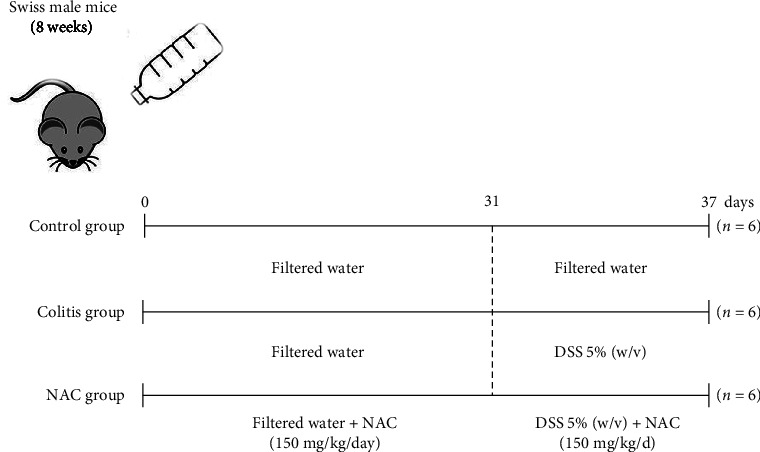
Experimental design of dextran sulfate sodium (DSS)-induced colitis and pretreatment with *N*-acetylcysteine (NAC). The mice in the NAC group received, for 30 days, 150 mg/kg/day of NAC dissolved in drinking water. On the 31^st^ day, colitis was induced by administering 5% DSS (w/v) dissolved in drinking water for 7 days, in all but the control group. The administration of NAC was continued in the NAC group.

The animals were divided into three treatment groups, with six animals each: control, colitis, and colitis + NAC (NAC group). Prophylactic and therapeutic administration of 150 mg/kg/day of NAC was performed for 30 consecutive days. From the 31^st^ day onward, colitis was induced for 7 days by addition of 5% (weight/volume (w/v)) DSS in the drinking water. Supplementation of the NAC group was continued for a total intervention time of 37 days. The previous and simultaneous administration of NAC into the DSS-induced colitis in mice aims to evaluate the effects of NAC before the onset of an acute colitis, to mimic the remission phase of the individual with IBD in continuous use of an antioxidant, aiming to evaluate the protective effect of NAC. Glycemia was evaluated at the beginning of the experiment (day 1), before (day 31), and after the induction of colitis (day 37; Figure 1). A dose of NAC at 150 mg/kg/day was selected based on our previous investigation of its hepatotoxicity [12]. A comprehensive review was held to establish safe dosage ranges. It is essential to highlight that NAC is orally administered and subsequently delivered to the liver via the portal vein, where it undergoes hepatic metabolism. In this context, previous studies have demonstrated that NAC exhibits acute or subacute toxicity in mice at doses of 600 and 2,000 mg/kg/day, respectively. It is worth noting that NAC, being highly soluble, is primarily eliminated through urine, with 13%–38% of the ingested NAC from the previous day being excreted within the first 24 hr [18]. Moreover, in our group's review of NAC's effects on liver tissue, positive outcomes on redox and inflammatory status were observed with doses ranging from 100 to 300 mg/kg/day, without any adverse effects [12]. Thus, based on these findings, we have justified our selection of 150 mg/kg/day as the preventive and therapeutic dosage.

### 2.5. Euthanasia, Blood Collection, and Preparation of Tissue Homogenates

On the 38^th^ day, after a 12hr overnight fast, the mice were anesthetized (ketamine, 100 mg/kg, and xylazine, 15 mg/kg, i.p.) and subjected to blood collection by cardiac puncture. After ventricular perfusion with 2% heparin solution, the aorta was sectioned, and the colon, liver, spleen, and kidneys were dissected. All organs, except spleen, were placed in liquid nitrogen and immediately stored at −80°C. Then, the tissue homogenates were prepared with RIPA buffer and protease inhibitor cocktail (one tablet for 50 mL of RIPA buffer) and centrifuged at 19,600 × *g* for 20 min at 4°C. The supernatant was stored at −80°C.

### 2.6. Histological Analysis

Colon fragments were fixed in Bouin solution and subsequently immersed in increasing concentrations of ethanol (70%, 80%, 90%, and 100%), before their embedding in paraffin. After processing, histological sections of 5 *μ*m thickness were prepared and stained with hematoxylin and eosin (HE) to evaluate cell microstructures. The stained slides were photographed using an optical microscope connected to a DP70 digital camera system and subsequently evaluated.

### 2.7. Oxidative Stress Biomarkers

SOD activity was quantified using the SOD Assay Kit according to the manufacturer's instructions. The absorbance was read at 450 nm in a spectrophotometer and the results expressed in units per milligram protein. Hydrogen peroxide (H_2_O_2_) was determined according to an established protocol, and the results read at 610 nmol and expressed as nanomoles per milligram protein [19]. The levels of CAT were monitored at 240 nm, according to the colorimetric method, previously described and was expressed in international units per milligram protein [20] Total glutathione (GSHt) and oxidized glutathione (GSSG) were determined according to an earlier reported method, with slight modifications [21]. Reduced glutathione (GSH) levels were calculated, according to the following equation:

(1) $$\begin{array}{c} GSHt=GSH–\left[2\times GSSG\right], \end{array}$$

and their ratio (GSH/GSSG) was calculated. GSH and GSSG levels were expressed in nanometers per milligram protein. Tissue protein levels were determined using the Bradford assay [22]. Malondialdehyde (MDA) levels were measured using reversed-phase ion-pair HPLC with ultaviolet detection at 270 nm and expressed as nanomole MDA per milligram tissue [23].

### 2.8. Inflammatory Biomarkers

Tissue cytokine levels (TNF-*α* and IL-10) were determined by enzyme-linked immunosorbent assay (ELISA), using the PeproTech® kit (PeproTech Brasil FUNPEC, Ribeirão Preto, SP, Brazil) according to the manufacturer's instructions. The absorbance reading was performed at 450 nm in an ELISA plate reader, and the results were expressed in picograms per milligram protein.

Myeloperoxidase (MPO) activity was measured by adapting the previously proposed method [24], and results were expressed in units per milligram protein. One unit of MPO was defined as the amount of enzyme required to decompose 1 *μ*mol H_2_O_2_.

### 2.9. Statistical Analysis

Data analysis was performed using the statistical software GraphPad® Prism (version 5.0) for Windows (San Diego, CA, USA), adopting *α* = 0.05. To verify the normal distribution, the Shapiro–Wilk test was used. Parametric variables were assessed using unidirectional paired analysis of variance (ANOVA), followed by a Tukey test with a Bonferroni correction for comparisons between various groups. The Kruskal–Wallis test was used to assess nonparametric variables, and the corresponding post hoc analysis was performed. The results were presented as mean ± standard error of the mean (SEM) for those with a normal distribution and values as median and interquartile range for nonparametric variables.

## 3. Results

### 3.1. Histological Analysis of the Colon

**Figure 2:**
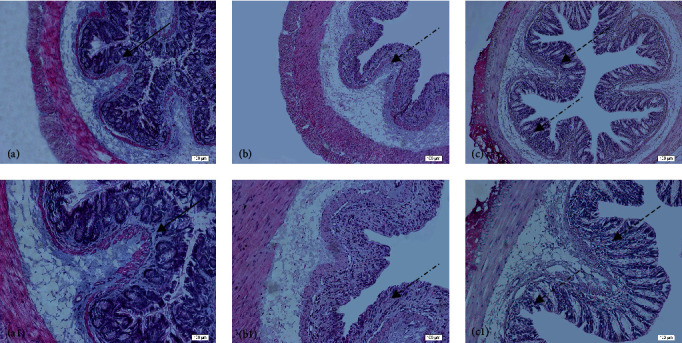
Histopathological analysis of the colon related to the experimental group: control group (a, a1), colitis group (b, b1), and *N*-acetylcysteine (NAC) group (c, c1). Representative photomicrographs of mouse colonic sections. Black arrow, preservation of the crypt structure; black dashed arrow, crypt destruction; and black dotted arrow, narrowing of the mucosal muscle. 10x magnification. Hematoxylin and eosin (HE) staining.

Histological evaluation showed changes in colon architecture related to colitis, including destruction, disarrangement, and shortening of the crypts, indicating that the administration of DSS induced UC in mice (Figure 2(b)), when compared to the control (Figure 2(a)). NAC administration partially restored the epithelial structure, as indicated by the intact areas in the histological section (Figure 2(c)).

### 3.2. Influence of Colitis and NAC Supplementation on Blood Glucose

**Figure 3:**
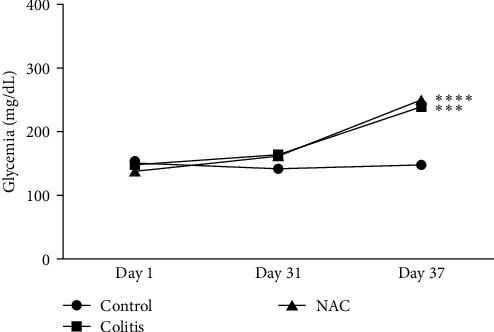
Glycemia, by group: control, colitis, *N*-acetylcysteine (NAC),  ^*∗∗∗*^*p* < 0.001,  ^*∗∗∗∗*^*p* < 0.0001, and Tukey test.

Hyperglycemia derived from chronic inflammation is considered one of the cardinal metabolic alterations in UC [25–27]. In the present UC model, a significant increase in blood glucose in the colitis group was observed, and NAC administration did not restore these levels (Figure 3).

### 3.3. Effects of Colitis and NAC on the Colon: Colitis and NAC Cause Redox Imbalance, and NAC Stimulates Inflammation

**Figure 4:**
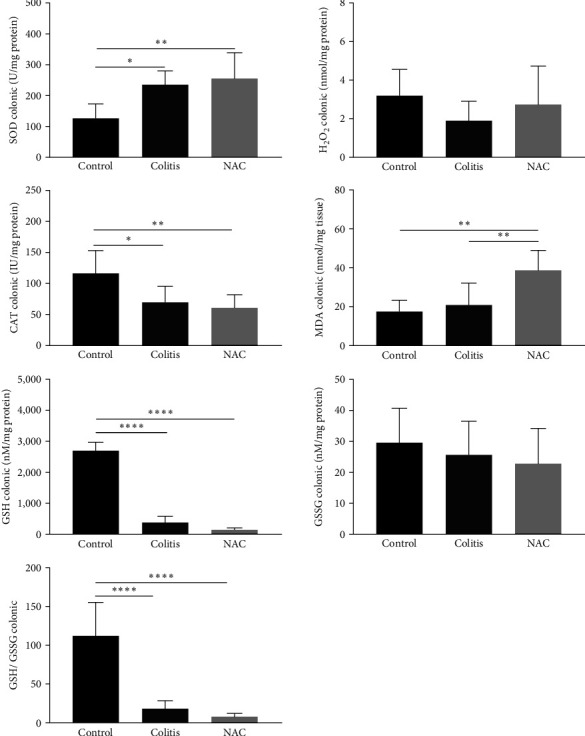
Colonic redox imbalance markers: superoxide dismutase (SOD) activity; hydrogen peroxide (H_2_O_2_) levels, catalase (CAT) activity, malondialdehyde (MDA) levels, reduced glutathione (GSH) levels, oxidized glutathione (GSSG) levels, and the GSH/GSSG ratio by group: control, colitis, *N*-acetylcysteine (NAC);  ^*∗*^*p* < 0.05;  ^*∗∗*^*p* < 0.01,  ^*∗∗∗*^*p* < 0.001,  ^*∗∗∗∗*^*p* < 0.0001, and Tukey test.

**Figure 5:**
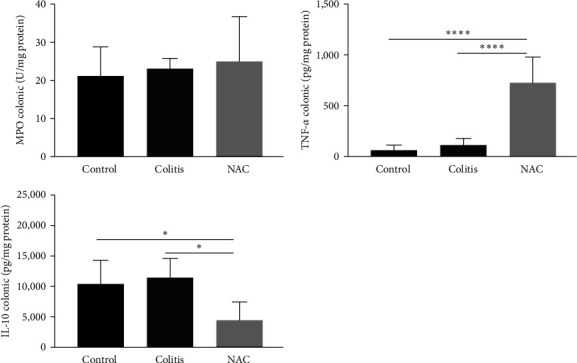
Colonic inflammatory markers: myeloperoxidase (MPO) activity, tumor necrosis factor alpha (TNF-*α*), and interleukin (IL)-10, by group: control, colitis, *N*-acetylcysteine (NAC),  ^*∗*^*p* < 0.05,  ^*∗∗*^*p* < 0.01,  ^*∗∗∗*^*p* < 0.001,  ^*∗∗∗∗*^*p* < 0.0001, and Tukey test.

The colitis group presented an alteration in antioxidant capacity versus the control group (increased SOD, decreased CAT, GSH, and GSH/GSSG ratio; Figure 4). NAC did not restore the antioxidant capacity and showed pro-oxidant (increased MDA levels) and pro-inflammatory (increased TNF-*α* and decreased IL-10) effects on this tissue (Figures 4 and 5), indicating its pro-oxidant and pro-inflammatory action on the colon.

### 3.4. Effects of Colitis and NAC Treatment on the Liver: Colitis Caused Redox Imbalance and Decreased Anti-Inflammatory Protection in the Liver, and Oral NAC Aggravated These Imbalances

**Figure 6:**
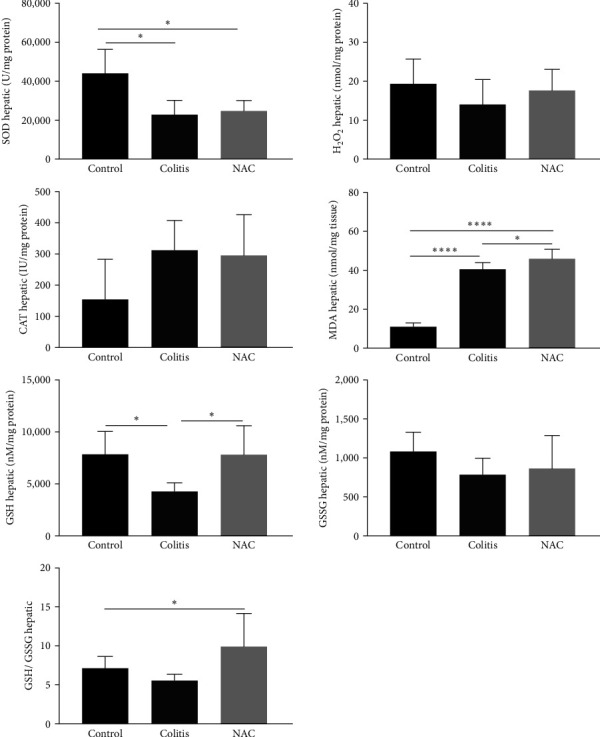
Liver redox imbalance markers: superoxide dismutase (SOD) activity, hydrogen peroxide (H_2_O_2_) levels, catalase (CAT) activity, malondialdehyde (MDA) levels, reduced glutathione (GSH) levels, oxidized glutathione (GSSG) levels, and the GSH/GSSG ratio, by group: control, colitis, *N*-acetylcysteine (NAC),  ^*∗*^*p* < 0.05,  ^*∗∗*^*p* < 0.01,  ^*∗∗∗*^*p* < 0.001,  ^*∗∗∗∗*^*p* < 0.0001, Tukey test (GSH, GSSG, and MDA), and Kruskal–Wallis (SOD, H_2_O_2_, CAT, and GSH/GSSG).

**Figure 7:**
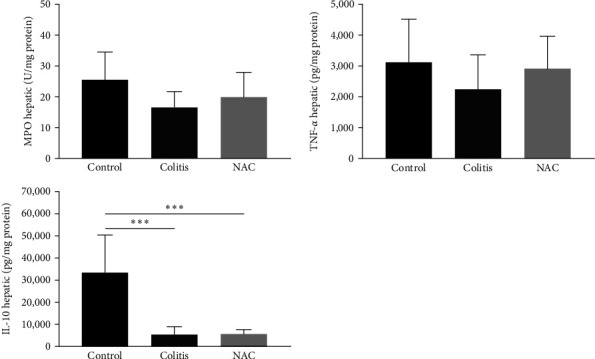
Hepatic inflammatory markers: myeloperoxidase (MPO) activity, tumor necrosis factor alpha (TNF-*α*), and interleukin (IL)-10, by group: control, colitis, and *N*-acetylcysteine (NAC).  ^*∗∗∗*^*p* < 0.001, Tukey test (TNF-*α* and IL-10), and Kruskal–Wallis (MPO).

Colitis caused lipid peroxidation (LP; increased MDA levels) and changes in antioxidant defense (decreased GSH levels) and inflammation profile (decreased IL-10 levels; Figures 6 and 7). NAC restored hepatic GSH and the GSH/GSSG ratio but not IL-10 and worsened LP in this organ, indicating a pro-inflammatory effect (Figures 6 and 7).

It was observed that SOD activity and GSH levels were reduced, and MDA was increased in the colitis group, compared to the control. However, NAC restored GSH and increased the GSH/GSSG ratio (vs. the control group). On the other hand, NAC worsened cellular damage (MDA levels), confirming the conflicting effect of NAC on redox imbalance (Figure 6). No other alteration was observed in this organ.

### 3.5. Effects of Colitis and NAC Treatments on the Kidney: Colitis Did Not Cause Oxidative or Inflammatory Damage in Kidney, and NAC Presented Conflicting Results

**Figure 8:**
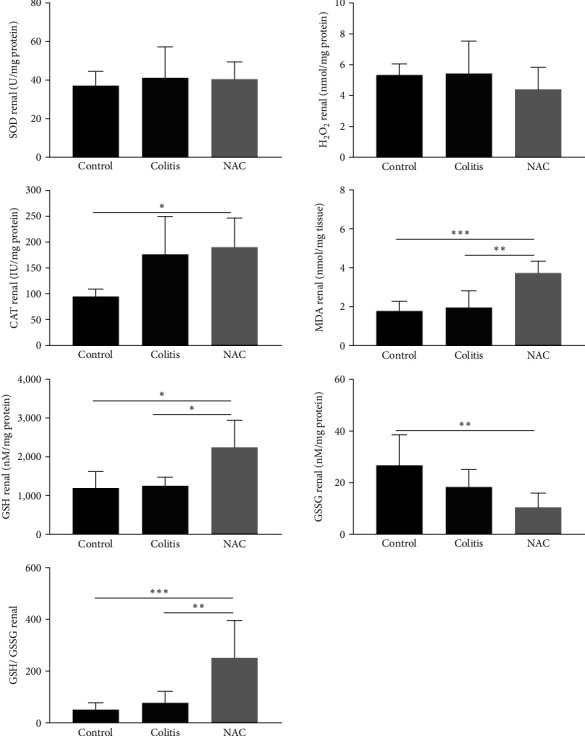
Renal redox imbalance markers: superoxide dismutase (SOD) activity, hydrogen peroxide (H_2_O_2_) levels, catalase (CAT) activity, malondialdehyde (MDA) levels, reduced glutathione (GSH) levels, and oxidized glutathione (GSSG) levels, by group: control, colitis, and *N*-acetylcysteine (NAC).  ^*∗*^*p* < 0.05,  ^*∗∗*^*p* < 0.01,  ^*∗∗∗*^*p* < 0.001, and Tukey test.

**Figure 9:**
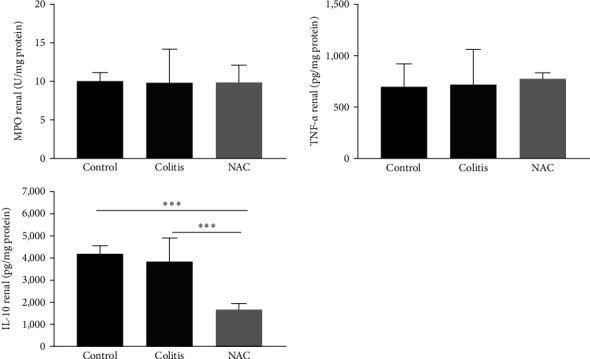
Renal inflammatory markers: myeloperoxidase (MPO) activity, tumor necrosis factor alpha (TNF-*α*), and interleukin (IL)-10, by group: control, colitis, and *N*-acetylcysteine (NAC).  ^*∗*^*p* < 0.05 ^*∗∗*^*p* < 0.01,  ^*∗∗∗*^*p* < 0.001, and Tukey test.

Colitis did not cause oxidative or inflammatory damage in the kidney. By contrast, although NAC significantly stimulated CAT activity and increased GSH and decreased GSSG levels, consequently improving the GSH/GSSG ratio, its administration leads to increased MDA levels (vs. the control and colitis groups) and decreased anti-inflammatory capacity in the kidney, by reducing IL-10 levels (Figures 8 and 9).

## 4. Discussion

In this research, we used DSS 5% (w/v) to induce UC, in mice, and to investigate EIM in the liver and kidney. DSS is a high-molecular-weight (40,000 Da) phosphate polysaccharide that contains up to three sulfate groups per glucose molecule. It acts directly on the colon, causing epithelial lesions in its mid-distal portion, which are responsible for the characteristic signs and symptoms of colitis in humans [28, 29]. As it is a molecule of high molecular weight, it does not cross the cell membrane and is poorly absorbed, its action being limited to the colon, thus allowing damage caused by disease *per se* to other organs to be investigated [30].

The histological changes mentioned above demonstrate that DSS effectively induced UC. Additionally, this experimental model was able to induce redox imbalance and modification of the inflammatory pannel in liver, but not in kidney. Despite NAC pretreatment had improved some markers of antioxidant and anti-inflammatory, negative effects were observed such as: (1) shortening of the colon, similar to that found by Kim et al. [31], studying GPx1−/− × Cat−/− mice fed with 400 *µ*L of NAC (40 nM) before (3 days) and during (4 days) colitis induction by DSS (3% w/v); (2) membrane damage in all organs studied (identified by MDA levels); (3) hyperglycemia; and (4) a decreased in anti-inflammatory power, discouraging the long-term use of this supplement.

These results in liver and kidney, despite having been obtained in an animal model that mimics UC, shed light on the use of alternative therapies or not in individuals with IBD and who also present associated diseases, such as diabetes, liver, and kidney diseases, since NAC had shown to play a harmful role on markers that reflect metabolic and systemic integrity. This negative effect of NAC had been shown before in others studies [32, 33] and can be explained by its pro-oxidant power, and, because of this property, has been tested to induce death in cancer cells [34, 35].

### 4.1. Effects of DSS and NAC on the Colon

Redox imbalance in the colon, characterized by the reduction of antioxidant defenses and increased levels of pro-oxidant molecules, is involved in the maintenance and progression of chronic inflammation in UC [3, 36–38]. In this context, anti-inflammatory and antioxidant molecules, such as NAC, would be expected to mitigate the symptoms of IBD.

It was observed that colitis, despite increased levels of SOD, which were probably due to increased levels of superoxide radical (O_2_^·−^) production, decreased CAT activity and the GSH/GSSG ratio and increased LP without raising H_2_O_2_ levels. This may be due to the increase in the Fenton reaction and consequent production of hydroxyl radicals (^·^OH) by Fe^2+^, which indiscriminately oxidizes phospholipids and intercellular junctions, causing damage to the intestinal barrier [39]. UC pathophysiology is also characterized by high levels of pro-inflammatory cytokines, secreted by cells of the innate immune system and activated T lymphocytes, and a decrease in anti-inflammatory cytokines [2].

The compensatory response of the body to the increased levels of reactive oxygen species (ROS) in the circulation is a typical physiological process in IBD pathophysiology, highlighting the redox imbalance in both cells and mucosa. However, the expected prophylactic and therapeutic administration of the anti-oxidation NAC did not occur, negatively influencing these biomarkers. These findings differ from a previous study from Amrouche-Mekkioui and Djerdjouri [40]. Amrouche-Mekkioui and Djerdjouri [40] identified a significant improvement in the antioxidant capacity (SOD, CAT, and GSH) after oral supplementation of NAC (150 mg/kg/day) for 45 days in a DSS-induced colitis model (5%–500% kDa). Some differences between the two assays may justify the different results, such as the higher molecular weight of the DSS, as well as the model of colitis induction and treatment with NAC chosen by the authors being 3 cycles of DSS/10 days of free water.

The exacerbated inflammatory process in the colon is responsible for the symptoms, complications, and clinical progression of the disease. Neutrophil infiltration into the *lamina propria* is a common finding in the symptomatic phase. The measurement of MPO, an enzyme secreted from the granules of these cells of the innate immune system, can be performed to estimate this inflammatory activity. In this work, DSS 5% (w/v) did not cause alterations in MPO levels, indicating the absence of pronounced release of this enzyme from the neutrophil granules.

In this study, the oxidative injury caused by colitis preceded the inflammatory damage, once the levels of MPO, TNF-*α*, and IL-10 did not change. These findings are similar to those previously reported by our group [41], which administered DSS (2%) in rats and did not identify changes in the inflammatory pattern in the animals. On the other hand, Amrouche-Mekkioui and Djerdjouri [40] observed inflammation by increasing the activity of MPO and pro-inflammatory cytokines, including TNF-*α*, in addition to oxidative damage (increased MDA).

Unexpectedly, however, consistent with pro-oxidant effect of NAC in others studies [32–35], oral supplementation of NAC caused activation of inflammatory pathways and impaired the anti-inflammatory responses, as evidenced by the increase in TNF-*α* levels and decrease in IL-10, as well as macroscopic shortening of the colon. In contrast to the findings of the present study, Amrouche-Mekkioui and Djerdjouri [40] demonstrated that NAC decreased the production of inflammatory and LP markers (MPO and MDA). Moura et al. [41] observed similar effects in a mild colitis model (DSS 2%) but did not observe changes in cytokine levels.

The pro-oxidant and pro-inflammatory actions of NAC are an unusual finding, given its antioxidant role described in the literature, which is attributed to its sulfhydryl (–SH) moiety, and that it mainly provides cysteine for the synthesis of GSH [11, 42]. It is likely that the low concentration of cytosolic acylase I (an enzyme responsible for producing cysteine via a deacylation reaction) in the colon has limited activity in the inflammatory scenario [43, 44].

Although NAC and other thiols, such as GSH, are considered excellent antioxidants, the literature reports that under the influence of certain conditions and factors, they can be subjected to thiol oxidation, which generates thiyl radicals. In the presence of oxygen and metals (Cu^2+^ and Fe^2+^), this results in the formation of ^·^OH and the O_2_^·−^, thus implying oxidative damage to biological systems [11, 32]. A similar effect occurs with the stilbene resveratrol [45, 46] and ascorbic acid [47].

In a study on human leukemia cells induced by cadmium, low doses of NAC presented an antagonistic action, intensifying the cytotoxic effect of this metal and behaving as a pro-oxidant. It was suggested that NAC can be reduced to hydrogen sulfide in mitochondria and produce H_2_O_2_ in the presence of oxygen [35]. However, this hypothesis requires further investigation and corroboration. A similar effect was reported with NAC in the presence of Cu^2+^ [12]. Additionally, a long-term treatment with a low dose of NAC increased the expression of pro-inflammatory cytokines [48].

However, one cannot ignore the histological improvement observed with the use of NAC, which, from a clinical perspective, may reflect complete healing [42]. In humans, histological remission has been identified as a promising tool and target to guide the treatment of patients with IBD, given its association with better overall clinical outcomes when compared to endoscopic remission [49, 50].

Therefore, at the colonic level, there was a loss of antioxidant capacity caused by colitis, and the prophylactic and therapeutic administration of NAC had no influence on this. However, in an unusual way, NAC exhibited pro-oxidant and pro-inflammatory behavior on colon, suggesting this supplementation is not safe to IBD.

This ambiguous effect of NAC can be observed in more detail when we analyze Table 1. In this table, we find 17 studies in a murine model, and 2 clinical trials in humans, that use NAC as a pretreatment or treatment of IBD.

In animal models, we can see that there is a great variation in the model of treatment (acute colitis–1 day of induction–to chronic colitis–204 days of induction by cycles), dose (0.00000026 mg–500 mg/day) and time of use of NAC (hours after colitis induction to 204 days).

Another relevant information obtained by analyzing Table 1 is that most of the studies observed an improvement in the microscopic and macroscopic pattern of the lesions, and a reduction in the inflammatory and oxidative profile, compared to the no-treated group. However, some negative effects stand out, such as causing colon shortening [31] and increased inflammation [31, 57] (like our findings) and promoting weight loss [31].

By the other side, studies in humans observed that patients treated with NAC noticed an improvement in the inflammatory pattern [17, 64] and a reduction in the endoscopic activity of the disease. However, the difference between the doses/time used (2.4 g/day for 4 weeks vs. 800 mg/day for 16 weeks) calls attention, making it difficult to compare the effect between the studies.

### 4.2. Hepatic Repercussions of DSS and NAC

The disruption of all intestinal barriers resulting from oxidative stress and the chronic inflammatory process of UC facilitates the systemic diffusion of luminal antigens and bacterial products and the consequent appearance of EIM. Considering its intimate connection with the colon, the liver is an easy target for the occurrence of inflammatory responses, fibrosis, genotoxicity, and hepatocellular damage [7].

In this context, liver involvement after induction of UC has been demonstrated in animal models using DSS [30, 65]. In this work, it was observed that colitis caused a prominent reduction in hepatic antioxidant capacity (decreased levels of SOD and GSH) and tissue damage, evidenced by the significant increase in LP, as measured by MDA levels, although no changes in H_2_O_2_ levels were observed. These findings corroborate reports from the literature regarding liver manifestations resulting from severe colitis (DSS 5%). In the induction model with 5% DSS for 5 days, a significant reduction in antioxidant capacity (SOD, CAT, GPx, and GSH) was observed, accompanied by an increase in H_2_O_2_ levels [40]. Despite the single use of MDA, as a marker for lipid oxidation, the significantly elevated levels observed in comparison to the control group, coupled with the diminished antioxidant capacity, indicate the presence of oxidative damage in hepatic cells. However, it should be noted that these biomarkers alone are insufficient to definitively confirm clinical liver injury, which is typically established through the measurement of transaminase levels.

Furthermore, it has been demonstrated with histological evidence that 3% DSS also causes hepatic damage, such as liver degeneration and diffuse necrotic lesions, via an increase in tissue LP (increased MDA) and reduced antioxidant defense (GSH, SOD, and CAT) [66].

The use of NAC, although it did not affect SOD and CAT activities, prominently increased the levels of GSH and the GSH/GSSG ratio. This result can be attributed to the abundance of cytosolic acylase I at the hepatic level, which, through a deacylation reaction, facilitates the efficient delivery of cysteine for GSH synthesis [36, 37]. However, it did not have the expected prophylactic and therapeutic benefit from NAC; on contrary, it caused hepatocellular damage (increased MDA), indicating a pro-oxidant activity.

NAC also presents conflicting results on the liver redox imbalance in diseases other than IBD. In an animal model of steatohepatitis, no influence of NAC (150 mg/kg/day) on the activities of SOD and CAT was observed [67]. In a fenitrothion-induced hepatotoxicity model, it was demonstrated that NAC (at 50 and 200 mg/kg/day, respectively) improved the redox imbalance by increasing the levels of GSH, SOD, CAT, and GPx. In contrast to this study, it also led to a reduction in MDA levels [68]. It is important to emphasize the lack of studies in the literature assessing the hepatic repercussions of NAC in UC model, which makes a more robust comparison impossible.

The repercussion of colitis on the hepatic inflammatory profile was investigated, through MPO activity and TNF-*α* and IL-10 levels. It was observed that colitis did not interfere with the requirement of MPO activity and TNF-*α* levels, indicating that these inflammatory markers were not activated or deactivated and that other cytokines not investigated (IL-6 and IL-1*β*, e.g.) may be involved. However, colitis caused a decrease in IL-10 levels, thus influencing the anti-inflammatory response of this organ. In contrast, several studies investigating hepatic tissue in colitis induction models with 3%, 4%, and 5% DSS reported increased levels of TNF-*α* and/or MPO activity and similarly to our work reduced levels of IL-10 [30, 66, 69, 70]. On the other hand, an experimental study that induced acute colitis with 2.5% DSS for 5 days observed increased TNF-*α* levels and maintenance of IL-10 levels of the livers of these animals [71].

The NAC treatment, in turn, had no effect on MPO activity and TNF-*α* and was not able to normalize the IL-10 levels. In agreement with the present study, a previous study did not report TNF-*α* level alterations resulting from the use of NAC (160 mg/kg/day) in an ischemia-reperfusion model [72]. In contrast to the findings mentioned earlier, a different study on liver toxicity reported a decrease in TNF-*α* levels in the group treated with NAC (160 mg/kg/day) [68]. Similarly, in a model of alcohol-induced liver damage, pre-treatment with NAC at various doses (75, 150, and 300 mg/kg) effectively prevented cell damage, while post-damage treatment exacerbated liver injury, increased lipid peroxidation, and showed limited impact on GSH levels and TNF-*α* expression [65].

According to Table 1, only two studies investigated liver damage, both studies in animals [41, 62]. Although the authors did not observe negative or positive effects, in a previous study carried out by our group Moura et al. [41] observed that NAC combined with lipoic acid increased markers of liver injury (aspartate aminotransferase (AST) and alanine aminotransferase (ALT)) and TNF-*α*, and significantly decreased IL-10, demonstrating the pro-oxidant effect of these two supplements combined.

Thus, according to the present results, colitis can cause hepatocellular damage, and the prophylactic and therapeutic use of NAC can have a dual effect: on the one hand, restoring tissue levels of GSH and, on the other, worsening LP. Thus, by observing colon and liver together, we can ratify the nonsafety of using NAC in IBD.

### 4.3. Renal Repercussions of DSS and NAC

EIM involving the kidney are under investigated, although cases of renal injury have been identified in humans [73, 74] and an experimental UC model [75].

A study conducted by Ranganathan et al. [75, 76] demonstrated the link between the colon and the kidney in DSS-induced UC, which caused acute kidney damage and increased levels of cytokines and chemokines, such as TNF-*α*, IL-6, IL-1*β*, and monocyte chemotactic protein (MCP-1), in contrast to this work, which did not observe changes in the renal oxidative or the inflammatory profile of the intestinal disease [76].

By contrast, prophylactic and therapeutic administration of NAC significantly increased the activity of CAT and GSH, as well as the GSH/GSSS ratio, and reduced the levels of GSSG and H_2_O_2_. This suggests that, even in the presence of colitis, NAC was effective in recruiting CAT and efficient as a cysteine provider for later formation of GSH, given the abundant amount of acylase I in the renal tissue, thus replenishing the endogenous antioxidant reserve in the kidney [43, 44]. Similarly, Nogueira et al. [77] demonstrated that 8 weeks of NAC supplementation in drinking water (600 mg/L) had a protective effect against kidney injury induced by diabetes, improving antioxidant defense by increasing the activity of CAT and GSH [77]. Nouri and Heidarian [78] also highlighted the antioxidant role of NAC (100 mg/kg) via stimulation of CAT and SOD activity in their model of kidney injury induced by diclofenac.

These findings bring new answers to the study of renal alterations in IBD, since, as can be seen in Table 1, only Shi et al. [62] evaluated renal markers in animals with induced colitis. It should be noted that these authors evaluated urea and creatinine, considered late markers of renal dysfunction, while we evaluated oxidative damage and inflammatory and anti-inflammatory cytokines, which are altered in early trauma situations.

Our findings differ from studies that evaluated the use of NAC as a treatment in different models of renal damage. Kondakçi et al. [79] observed that 1 g/kg/day of NAC for 6 weeks, in a model of renal oxidative stress induced by thiolactone homocysteine, reduced tissue levels of ROS and MDA. Yao et al. [80], in turn, found that oral administration of 150 mg/kg/day of NAC for 7 consecutive days, by gavage, during nephrotic syndrome induction, resulted in a reduction in the levels of TNF-*α* and IL-6, but the authors did not measure IL-10 concentrations.

In summary, UC induced by DSS did not provoke any manifestation in the kidney. On the other hand, prophylactic and therapeutic administration of NAC had various effects on the antioxidant and inflammatory kidney profile; therefore, its renal action associated with IBD should be better evaluated, especially in those who already have altered kidney function.

Again, despite some beneficial antioxidant effects of NAC consumption found in this study, the negative alterations that may cause irreversible oxidative and inflammatory damage in the colon, liver, and kidney confirm the nonsafety of the prophylactic use of this antioxidant in models of induced colitis. We suggest that future research investigating antioxidant therapies in humans should consider assessing markers that evaluate the integrity and functionality of other organs. This may include measurements of serum transaminases, creatinine, and urea, among other widely used clinical indicators.

According to our research, this is the first study investigating the action of NAC on kidney injury in experimental colitis (Table 1).

## 5. Conclusions

In summary, DSS-induced colitis caused histological and oxidative alterations in the colon and redox imbalance in the liver (indicating EIMs), but not in the kidney. Regarding the prophylactic and therapeutic use of NAC, conflicting outcomes were observed. NAC, despite restoring some markers of antioxidant defense, increased LP and decreased anti-inflammatory protection in all tissues, indicating its pro-oxidant effect.

This study demonstrates that the prophylactic administration of NAC seems not to be adequate in DSS-induced colitis, once it affects the colon and other organs. Additionally, new questions have evolved: The use of NAC alone for therapeutic purposes at the colonic and extraintestinal levels would be more satisfactory and effective? Other models of colitis induction cycles (such as the active/remission phase that occurs in humans) would be better for evaluating the occurrence of EIMs and more comparable with humans (translationally).

Only through the interest of the scientific community, it will be possible to evaluate the molecular signaling pathways involved not only in the colon but in various organic systems of individuals with IBD, since health is wide and involves adequate systemic homeostasis.

In addition, even though this work is proposing to answer two questions raised by the European Crohn's and Colitis Organization, at least 28 of them remained open or with incomplete answers. Further studies are urgently required.

## Figures and Tables

**Table 1:** Effect of *N*-acetylcysteine (NAC) in experimental colitis and inflammatory bowel disease randomized clinical studies.

Author	Inflammatory bowel disease study	NAC effect			
		Colon	Liver	Kidney	Others
Animal model					
Ardite et al. [51]	Animals: male sprague-dawley rats weighing 200–250 g; C.i.: 30 mg of TNBS—once; NAC treatment: 40 nM (water (w/v)) for 4 hr after c.i.	↑ Mucosal GSH levels; improved histologic score	NT	NT	NT

Nosál'ová et al. [52]	Animals: wistar rats; C.i.: 4% (v/v) acetic acid (i.r.), once; NAC treatment: 20, 40, 100, or 200 mg (i.r.), before c.i., 100 mg (i.r.) after c.i.	NT	NT	NT	Pretreatment: prevented weight loss (dose-dependently)

Seril et al. [53]	Animals: C57BL/6J mice; C.i.: 0.7% DSS water (w/v)/7 days, for 12 cycles: 7 days DSS—10 days water free; NAC treatment: 2x/day–0.2%/day (∼200 mg/kg) NAC in diet (45 mg iron/kg add in AIN76A diet) during all cycles of c.i.	↓ UC index; ↑ apoptosis; did not alter inflammatory cells	NT	NT	Did not alter weight body

Akgun et al. [5]	Animals: swiss rats; C.i.: 4% (v/v) acetic acid (i.r.), once; NAC treatment: 20 mg/kg and 100 mg/kg (i.r and/or i.p.), for 2 or 7 days	NAC 100 mg/day for 7 days: ↓ inflammation (MPO levels) and nitrosative stress (↓iNOS); did not improve GSH levels	NT	NT	NT

Kurutas et al. [55]	Animals: wistar rats; C.i.: 4% (v/v) acetic acid (i.r), once; NAC pretreatment: 500 mg/kg/day (i.p.), 1 hr before c.i.	↓ MPO activity; normalized SOD activity	NT	NT	NT

Cetinkaya et al. [56]	Animals: wistar rats; C.i.: 4% (v/v) acetic acid (i.r.), once; NAC pretreatment and treatment: 500 mg/kg (i.p. or i.r.), 1 hr before c.i. or 24 hr after c.i.	NAC 1 hr before c.i.: prevented the reduction of antioxidant defense (↑ GSH levels). NAC 24 hr after c.i.: ↓ macroscopic damage (NAC i.r. and i.p.); ↓ inflammation (MPO acitvity; NAC i.r. and i.p.); ↓ oxidative damage (MDA levels); prevented the reduction of antioxidant defense (↑ GSH levels - mainly NAC i.r. –, SOD and CAT activity)	NT	NT	NT

Damiani et al. [57]	Animals: wistar rats; C.i. 50 g/L of DSS in water/5 days; NAC treatment: 20 mg/kg (s.c.), 2x/day, during c.i.	Did not prevent macroscopic lesion score; prevent macroscopic damage; did not prevent oxidative damage (TBARS level)	NT	NT	↑ Leukocytosis

Gommeaux et al. [58]	Animals: TP53INP1-deficient mice and WT mice; C.i.: 3.5% DSS water (w/v)/7 days; NAC pretreatment and treatment: 10 mg/mL (water (w/v)) for 10 days before, during, and after c.i.	NT	NT	NT	Delayed diarrhea and rectal bleeding (TP53INP1 -deficient mice and WT mice); no change in the weight loss and mortality; inhibit tumorigenesis (WT mice)

You et al. [59]	Animals: balb/c mice; C.i.: 5% DSS water (w/v)/7 days; NAC treatment: 0.3 mL NAC (i.r.), during c.i.	Improved colon shortening; ↓ DAI score; ↓ histologic score; ↓ inflammation (↓ MPO activity, IL-1*β* and TNF-*α* levels); ↓ oxidative damage (↓ ROS and MDA levels, and ↑ paraoxonase 1 and GSH activities)	NT	NT	Improved ↓ weight body

Amrouche-Mekkioui and Djerdjouri [40]	Animals: NMRI mice; C.i.: 5% DSS water (w/v), for 3 cycles: 5 days DSS—10 days water free; NAC treatment: 150 mg/kg (water) during all cycles of c.i.	Improved: ulcerative colitis score, histological score, clinical response, and mucosa thickness; ↓ inflammation (MPO activity) and oxidative damage (MDA and protein carbonyl levels, and iNOS activity); ↑ antioxidant defense (CAT and GSH activity); prevented mitochondrial dysfunction	NT	NT	NT

Uraz et al. [60]	Animals: wistar rats; C.i.: 4% (v/v) acetic acid/3 days (i.r); NAC treatment: 500 mg/kg/day (i.r), 5 min after c.i.	Prevented increased of wet weight; ↓ macroscopic and microscopic lesion scores; ↓ inflammatory activity (↓ MPO, IL-1*β*, IL-6, and TNF-*α*); prevented oxidative damage (↓TBARS); ↑ antioxidant defense (SOD and GSH);	NT	NT	NT

Kim et al. [31]	Animals: C57BL/6 WT and GPx1^−/−^ × Cat^−/−^ mice; C.i.: 3% (w/v) DSS water for 12 hr in a day/4 days; NAC pretreatment and treatment: 40 mM (gavage) for 3 days before c.i. and for 4 days during c.i.	Worst colon shortening (GPx1^−/−^ × Cat^−/−^ mice); promoted severe inflammatory changes and expression of pY-Stat3 (WT mice)	NT	NT	Promoted severe weight loss (GPx1^−/−^ × Cat^−/−^ mice).

Moura el al. [41]	Animals: wistar rats; C.i.: 2% DSS water (w/v)/5 days; NAC pretreatment and treatment: 100 mg/kg (diet), for 7 days before c.i. and during c.i.	Prevented histologic damage; normalized oxidative damage (H_2_O_2_, MDA, and GSH levels); did not alter inflammation (TNF-*α*, INF-*γ* and IL-10)	Did not alter weight body, oxidative damage, antioxidant defense or inflammation	NT	Prevented anemia and leukocytosis

Cha et al. [61]	Animals: C57BL/6 WT IDH2+/+ and knockout IDH2−/− mice; C.i.: 2% DSS water (w/v)/7 days; NAC pretreatment and treatment: 100 mg/kg (i.p.) for 3 days before, and during c.i.	Prevented colon shortening; improved histologic score; improved apoptosis; ↑ NF-*κ*B	NT	NT	NT

Shi et al. [62]	Animals: C57BL/6 mice; C.i.: 3% DSS water (w/v)/7 days; NAC treatment: 2 mg/kg (gavage), during c.i.	↓ DAI score; prevented colon shortening; improved histologic score; prevented oxidative damage (↓ MDA and ROS); normalized GPx activity; ↓ inflammation (↓ IL-1*β*, IL-6, MCP-1, and TNF-*α*)	No change was observed (AST and ALT–normal serum levels)	No change was observed (creatinine–normal serum levels)	↑ Body weight

Wang et al. [63]	Animals: C57BL/6N mice; C.i.: 3% DSS water (w/v)/7 days + HFCS; NAC pretreatment and treatment: 250 mg/kg (gavage), during c.i.	Prevented colon shortening	NT	NT	Prevented weight loss; ↓ inflammation (TNF-*α*, IL-6, and IL-1*β* serum levels)

Human studies					
Guijarro et al. [16]	Type of study: randomized, placebo-controlled pilot study; patients: 37 UC patients (18–70 years); NAC treatment: 2.4 g/day/4 weeks plus mesalamine				↓ Inflammation (serum IL-8 and MCP-1 levels)

Shirazi et al. [17]	Type of study: a randomized, double-blind controlled clinical trial; patients: 169 UC patients (18–75 years); NAC treatment: 400 mg–2x/day/16 weeks plus mesalamine	↓ Frequency of endoscopic relapse			↓ Inflammation (↓ fecal calprotectin, serum ESR, and hs-CRP levels)

ALT, alanine aminotransferase; AST, aspartate aminotransferase; CAT, catalase; c.i., colitis induction; DAI, disease activity index; DSS, dextran sulfate sodium; ESR, erythrocyte sedimentation rate; GPx, glutathione peroxidase; GSH, glutathione reduced; HFCS, high-fructose cornsyrup; hs-CRP, high-sensitive C-reactive protein; IDH, mitochondrial NADP+-dependent isocitrate dehydrogenase; IL, interleukin; INF-*γ*, interferon gamma; i.p., intraperitoneal; i.r., intrarectal; MCP-1, monocyte chemotactic protein 1; MPO, myeloperoxidase; NF-*κ*B, factor nuclear kappa B; pY-Stat3, anti-phosphotyrosine -Stat3; ROS, reactive oxygen species; TBARS, thiobarbituric acid reactive species; TNBS, 2,4,6-trinitrobenzenesulfonic acid; TNF-*α*, tumor necrosis factor; TP53INP1, tumor protein 53-induced nuclear protein 1; UC, ulcerative colitis; UC index (lesion severity, ulceration, hyperplasia, area of inflammatory involvement, and total histological score); v, volume; w, weight; WT, wild type.

## Data Availability

Data can be available on request through by correspondence author: fabiana.moura@fanut.ufal.br.
